# Pronounced mediastinal emphysema after restorative treatment of the lower left molar—a case report and a systematic review of the literature

**DOI:** 10.1007/s10006-022-01088-5

**Published:** 2022-06-10

**Authors:** Johannes Spille, Juliane Wagner, Dorothee Cäcilia Spille, Hendrik Naujokat, Aydin Gülses, Jörg Wiltfang, Paul Kübel

**Affiliations:** 1 Department of Oral and Maxillofacial Surgery, Christian Albrechts University, UKSH-Campus Kiel, Arnold-Heller-Straße 3, 24105 Kiel, Germany; 2 Department of Neurosurgery, University Hospital Münster, Münster, Germany

**Keywords:** Mediastinal emphysema, Dental treatment, Root canal treatment, Restorative Treatment, Complications

## Abstract

This case report presents an iatrogenic induced mediastinal emphysema after restorative treatment of the lower left second molar, aimed to highlight the potential life-threatening consequences, and providing diagnostics and treatment concepts of complicated dental induced emphysema based on literature review. A 74-year-old female patient was admitted to the emergency department due to a fall on her shoulder. Additional finding was a significant swelling of the face and neck. In the computer tomography of the head, neck, and thorax, a humerus fracture and pronounced soft tissue emphysema from the infraorbital region to the mediastinum was detected. The patient reported that she had been treated by her dentist 4 days earlier. The treatment had to be discontinued after beginning of a pronounced swelling. Other reasons for the emphysema could be excluded out on an interdisciplinary teamwork. The patient was monitored as an inpatient for 5 days and received intravenous antibiotic therapy. This case report shows the rare complication of pronounced mediastinal emphysema after root canal treatment. Emphysema should always be a differential diagnosis of soft tissue swelling and, in case of doubt, a general medical presentation should be made.

## Introduction

Mediastinal emphysema, also called pneumomediastinum, can be spontaneous as well as secondary and is the pathologic presence of free air enclosing mediastinal structures. The secondary mediastinal emphysema is associated with trauma and iatrogenic injury, which can occur after a dental treatment [1]. Emphysema as a complication of dental treatment is a rare complication that is often underestimated. It can occur in various treatments such as root canal treatment, professional teeth cleaning, tooth extraction, conservative, and prosthetic therapy [2–4]. Although iatrogenic emphysema is a very important differential diagnosis of facial and mediastinal swelling, it is rarely diagnosed by professional dentists and often played down. Whereby the spread of emphysema into the mediastinum can lead to life-threatening consequences, accurate diagnosis of emphysema must be made by clinical and radiological examination [2, 5]. There are several articles in the literature that address the causes of emphysema by dental treatment. This case report describes a pronounced mediastinal emphysema due to tooth preservation treatment in the molar region of the lower jaw, affecting the entire thoracic region and highlighted similar case reports based on literature search performed between 2010 and 2021, regarding their development, extent of disease, diagnostics, and treatment concepts.

## Case presentation

A 74-year-old female patient was admitted by an ambulance to the interdisciplinary emergency department of the University Hospital Schleswig–Holstein, Campus Kiel, after a fall on the right shoulder and on the face while walking with her dog. Anamnestically, the patient reported pain in the region of the right shoulder and the right hemithorax. In addition, there was a pronounced swelling of the face and neck, but this had already existed since dental treatment 4 days earlier. The treatment was discontinued after beginning of the swelling. After that, the patient was seen by her general practitioner. According to her medical history, she was given cortisone. Furthermore, the patient reported a hoarse voice immediately after dental practice, which was significantly regressive.

**Fig. 1:**
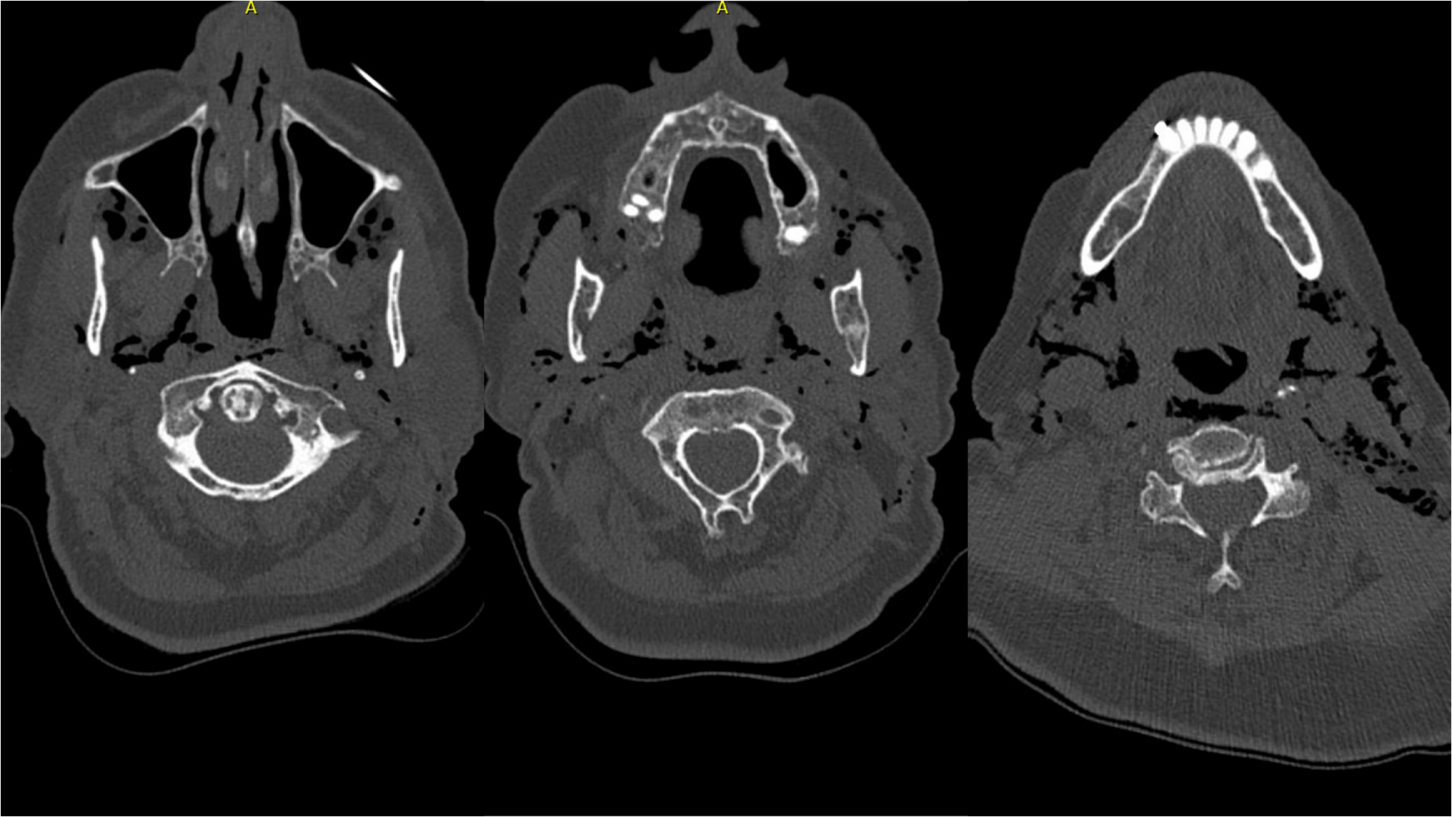
The spread of emphysema extends from the midface through the masseter loge to the mandibular rim. This was the initial computer tomography from the head until the neck. The emphysema was a secondary diagnosis

The initial computer tomography of the head and neck revealed prominent left cervical soft tissue emphysema. The emphysema extended to the prevertebral fat lamella and cranially into the Masseter’s lodge (Fig. 1). No fistula was demarcated from the oesophageal or tracheal regions. Furthermore, there was a questionable acute non-displaced fracture of the left nasal bone. There were no acute intracranial or cervical trauma consequences following her fall.

**Fig. 2:**
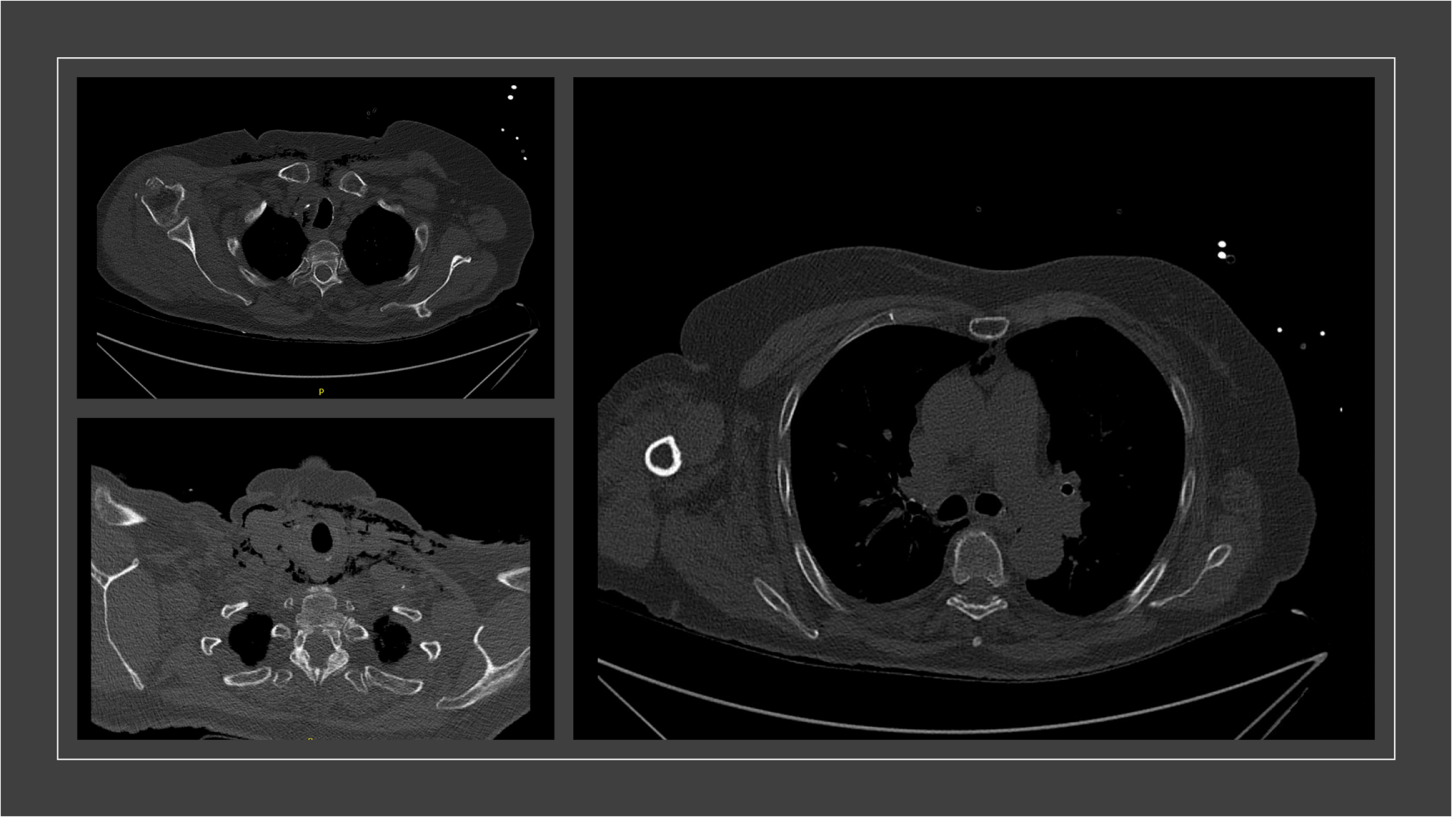
Following computed tomography of the thorax was able to diagnose further spread of emphysema. It extended from the neck to retrosternal and into the mediastinum

An X-ray examination (conventional X-ray and computer tomography) of the right shoulder showed a dislocated fracture of the right humeral head. Conventional X-ray examination of the right elbow, the right ribs, and thorax showed no fractures. Additionally, soft tissue emphysema extending from the cervical to the thorax with mediastinal emphysema was detected. A following computer tomography of the thorax (Fig. 2) could not definite a cause for the mediastinal emphysema. Only a slightly dislocated fracture of the 6th and 7th rib on the left ventral side could be detected. In addition, there was a small pleural effusion on the right side and a sintering of the thoracic spine, which appeared to be older.

To protect the shoulder fracture, a Gilchrist bandage was applied by the surgical trauma team. A further surgical treatment under general anaesthesia was planned in a few days.

Due to the pronounced emphysema, an oesophageal perforation was excluded by gastroscopy and a bronchoscopy was performed to exclude a cause in the tracheal and pulmonary region.

A clinical presentation to the department of otolaryngology did not reveal a focus on their specialty. The fracture of the nasal bone did not require further treatment. Clinically, only a small abrasion on the nasal bridge was evident.

Thus, the patient was monitored at the Clinic for Oral and Maxillofacial Surgery. The anamnesis and the clinical and radiological examination revealed that the cause of the pronounced soft tissue emphysema was a root canal treatment and restorative treatment of tooth 37 4 days earlier in a dental practice, during which air was insufflated into the surrounding tissue. The treatment started without any problems; immediately after air insufflation, swelling began to spread throughout the infraorbital region. The patient reported that previous dental procedures were performed without pathological abnormalities. The oral inspection revealed no wound or laceration.

On arrival, the patient showed no dyspnoea or respiratory distress. The vital signs showed no danger to life, blood pressure was 140/87 mm Hg, heart rate was 80 beats per minute, respiratory rate was 12 per minute, temperature was 36.6 °C, oxygen saturation was 99%, and Glasgow Coma Scale was 15. On clinical examination, the entire area of swelling appeared soft with noticeable crepitation; however, pain was denied. There was no severe redness, hyperthermia of the tissue, or other pathologic abnormalities. The patient reported taking only aspirin for routine treatment and to be healthy.

Laboratory control showed elevated leukocytes at 12.78 × 10^9/l and C-reactive protein at 85.5 mg/l. The patient was treated as an inpatient and monitored. The patient received intravenous antibiotic therapy with sulbactam/ampicillin 1.5 g three times daily and pain medication as needed. In the laboratory controls, the infection parameters showed regression, and the swelling also showed clinical improvement. Vital signs were without pathological abnormalities at all times. The patient could be discharged home after 5 days in a cardiac and pulmonary good general condition with oral antibiotics. In a follow-up examination 7 days later, the clinical examination showed no further pathological abnormalities.

## Literature review

A detailed review of the English literature from 2010 to 2021 was performed using the Pubmed database. The terms “dental”, “dental treatment”, “mediastinal emphysema”, “pneumomediastinum”, and “complications” were used for this purpose. Thirty-three articles could be found and evaluated and are summarised in Table 1. All patients presented a pronounced emphysema from the facial to the mediastinum.

**Table 1:** A comprehensive review of the literature from 2010 to 2021 was conducted through the PubMed database, using the research terms “dental”, “dental treatment”, “mediastinal emphysema”, “pneumomediastinum”, and “complications”. Developments of emphysema after different dental treatments are shown”

Reference	Tooth	Procedure	Treatment concept
Ocakcioglu et al. [1]	Third right mandibular molar	Extraction	Hospitalisation for four days, intravenous broad-spectrum antibiotics, gastroscopy
Kaliszewski et al. [3]	Third right maxillary molar	Extraction	Hospitalisation for five days, antihistamine drugs, gastro- and bronchoscopy, intravenous antibiotics
Rad et al. [5]	Second left mandibular molar	Extraction	The patient was monitored several hours, discharged with oral antibiotics, no computer tomography
Nozewski et al. [8]	Second right mandibular molar	Extraction	Hospitalisation for two days, intravenous antibiotics
North et al. [9]	First right maxillary molar	Extraction	Hospitalisation for five days, diphenhydramine intravenous, flexible laryngoscopy, ampi-cillin/sulbactam, and clindamycin intravenous; intubation, mediastinal drainage one day later because of deterioration of the symptoms. Amoxicillin oral for six days as outpatient
Liu et al. [10]	Third left mandibular molar	Extraction	Hospitalisation for seven days, intravenous antibiotics, amoxicillin/clavulanic acid
Akra et al. [14]	First right mandibular molar	Extraction	Conservative treatment after a whole day of observation
Cuccia et al. [17]	Second left mandibular molar	Extraction	Hospitalisation for 7 days, intravenous corticosteroids and antibiotics, discharged with oral antibiotics (3rd generation cephalosporins for 5 days)
Brzycki [25]	First left mandibular molar	Extraction	Hospitalisation for three days, intravenous antibiotics, no computer tomography
Afzali et al. [28]	Second left mandibular molar	Extraction	Hospitalisation for five days, intravenous antibiotics (Clindamycin, Ceftazidime)
Elia et al. [29]	Second rigth mandibular molar	Extraction	Hospitalisation for seven days, intravenous antibiotics
Picard et al. [30]	Third right mandibular molar	Extraction	Hospitalisation for four days, intravenous antibiotics, discharged with oral antibiotics for one week
Thompson et al. [31]	Third left mandibular molar	Extraction	Hospitalisation (unknown number of days), intravenous antibiotics, no computer tomography
Tay et al. [32]	All third molars	Extraction	Hospitalisation for five days, intravenous antibiotics (amoxicillin/clavulanic acid)
Isik et al. [33]	Third right mandibular molar	Extraction	Hospitalisation for seven days, intravenous antibiotics
Ozdemir et al. [34]	First left mandibular molar	Extraction	Hospitalisation for six days, intravenous antibiotics (vancomycin, meropenem, and gentamicin)
Ramnarine et al. [15]	Three teeth	Filling therapy	Conservative treatment after several hours of observation
Busuladzic et al. [21]	First left mandibular premolar	Filling therapy subgingival	Hospitalisation, mediastinal drainage, intravenous antibiotic: piperacillin/tazobactam, vancomycin, and clindamycin. Moxifloxacin oral for seven days as outpatient
Chien et al. [26]	First right mandibular premolar and molar	Filling therapy	Treatment as outpatient with amoxicillin/clavulanic acid oral for five days
Rawlinson et al. [35]	Upper left and lower right molar teeth	Filling therapy	Hospitalisation on an intensive care unit, esophagogram, intravenous antibiotics for 24 h
Nishimura et al. [36]	Unknown	Filling therapy	Hospitalisation (unknown number of days), intravenous antibiotics
Lee et al. [37]	First right mandibular premolar	Filling therapy	Hospitalisation for eight days, intravenous antibiotics (ampicillin/sulbactam and metronidazole)
Michel et al. [20]	Second left mandibular molar	Crown preparation	Intravenous ampicillin-sulbactam for 2 days, discharged with oral antibiotics (amoxicillin-clavulanate for 5 days)
An et al. [27]	First right mandibular premolar	Endodontic treatment	Hospitalisation, intravenous antibiotic: clindamycin, dexamethasone, acidophilus capsules for 24 h; esophagogram because of deterioration of the symptoms; ampicillin and metronidazole intravenous. Amoxicillin/clavulanic acid oral for four days as outpatient
Durukan et al. [16]	First right mandibular molar	Endodontic treatment	Hospitalisation for three days, intravenous antibiotics: metronidazole, ampicillin
Chen et al. [19]	Second left mandibular premolar	Endodontic treatment	Hospitalisation for five days, intravenous antibiotic: apicillin/sulbactam. Amoxicillin/clavulanic acid oral for 12 days as outpatient
Kim et al. [38]	First left maxillary molar	Endodontic treatment	Hospitalisation for five days, intravenous antibiotics
Lau et al. [4]	All teeth	Teeth polishing	Hospitalisation for four days, intravenous antibiotics
Bocchiliani et al. [39]	All teeth	Airflow procedure	Hospitalisation for four days, intravenous antibiotics
Mitsunaga et al. [40]	First left maxillary molar	Tartar removal with an Er: YAG laser	Hospitalisation, airway monitoring, bed rest, and prophylactic intravenous antibiotic therapy for five days
Wong et al. [41]	Third right mandibular molar	Coronectomy	Treatment as outpatient with analgetic drugs
Lee et al. [42]	Second right maxillary anterior tooth	Periimplantitis therapy	Hospitalisation for 13 days, intravenous antibiotics (cephalosporin and piperacillin/tazobactam) for seven days
Moschetta et al. [43]	Primary teeth	Devitalization	Hospitalisation three days, intravenous antibiotics

In 21 patients (63.6%), the dentists treated only the teeth of the lower jaw. In most cases, the molars were treated (76%). In 48.5%, teeth were extracted; in 51.5%, other dental treatments such as filling therapies, endodontic treatment, and crown preparation were done. Pneumomediastinum was detected with computer tomography of the thorax in 30 patients (90.9%). In five cases (15.2%), further examinations such as gastroscopy and bronchoscopy were performed. The treatment with antibiotics was heterogenous; however, 27 patients got an intravenous antibiotic therapy (81.8%). One patient was treated surgically.

In all cases, the patients evolved well with resolution of the mediastinal emphysema.

## Discussion

Emphysema—especially subcutaneous emphysema extending into the mediastinum—are rare complications following dental treatment but can lead to prolonged hospitalisation. Usually, patients have a good prognosis, and within a week, the symptoms should be significantly regressed. However, in rare cases, further surgical intervention, such as a tracheotomy to secure the airway or mediastinal drainage, is necessary [6]. Furthermore, this complication can lead to acute danger to life especially the airway is impaired and even intubation becomes necessary [7]. That is the reason why the patients’ vital parameters were monitored during the hospital stay. The patient and the attending physicians appeared to underestimate the potential danger of mediastinal emphysema previously. Nozewski et al. reported that the actual number of cases detected worldwide would be greatly underestimated and that there is a lack of knowledge about the complication after dental intervention [8].

Other life-threatening complications could be a pneumothorax, an infection as well as a mediastinitis, nerve damage, or cardiac problems [9, 10]. Moreover, in 20 to 30%, a patient has a foramen ovale, and embolism—which can result in neurotoxic effects—can occur as a life-threatening complication. Due to resorption of the emphysema into the cardiovascular system, the air could be immediately transmitted intracranially via the foramen ovale [11, 12]. Therefore, it seems to be necessary to treat patients with pronounced emphysema as inpatients and to ensure that the emphysema is sufficiently diminishing. Concerning this matter, antibiotic therapy should always be considered to avoid superinfection or even sepsis. Further studies recommend prophylactic antibiotic therapy with penicillin until the air has been absorbed naturally [13–15]. Due to hospital standards, the patient received a broad-spectrum antibiotic (ampicillin/sulbactam) intravenously during her inpatient stay and until the infect parameters were normal. This therapy was continued orally on an outpatient basis and stopped at the aftercare check-up 5 days later because the clinical examination was unobtrusive. Penicillin as a broad-spectrum antibiotic is reported in the literature as an adequate coverage of the buccal flora [11, 13].

During dental procedures, the use of high-speed drills as well as isolation with air nozzles is standard. Nowadays, iatrogenic subcutaneous emphysema in the face can be associated with these treatment steps even during minor dental procedures [16]. As soon as air has entered the soft tissue space, it can spread along the complex fascial planes and anatomic spaces of the face and neck into the mediastinum or retropharyngeal region. In this context, most of the iatrogenic emphysema is described by root canal treatments or tooth extractions in the mandibular and molar region, because of the anatomical connection to these spaces [17–19]. In addition, preparations and filling therapies of mandibular molar regions can also lead to these complications [20]. Although soft tissue emphysema is more likely as a result from treatments of mandibular teeth due to their anatomical relationships, nevertheless, emphysema can occur also during all dental treatments in the upper jaw by using air supply [21].

**Fig. 3:**
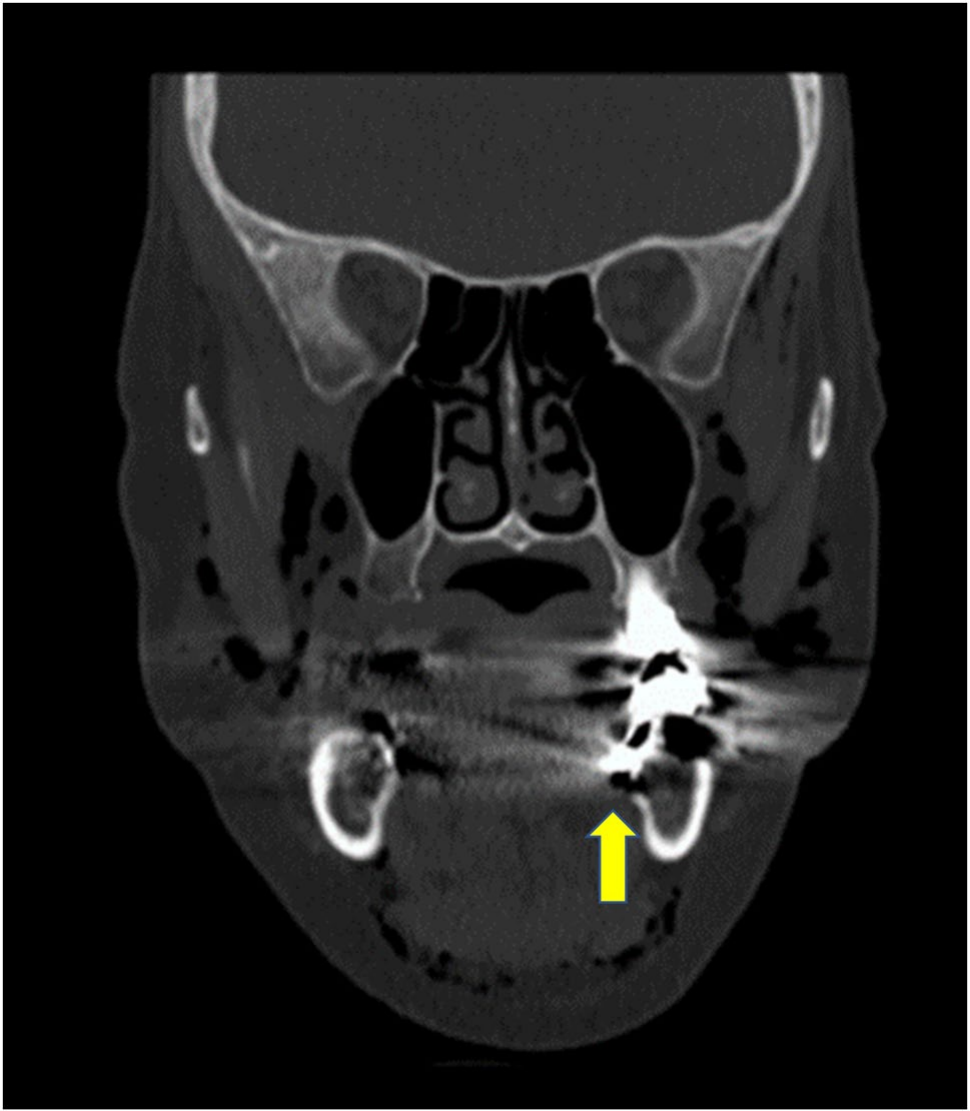
The lingual root canal of the lower left molar lays into the mouth base. The yellow arrow highlighted the entrance of the air into the cervical fascia and their lodges

In the literature are some studies describing apical inflammations of the second molars of the lower jaw which can lead to unusual abscesses. The spread of inflammation is explained by the lingual root, which can sometimes extend into the mouth base. This provides connections to the cervical fascia and their lodges [22, 23]. In the coronal slice of the computed tomography scan, the lingual root of tooth 37 can also be seen (Fig. 3). The root extends far lingually into the mouth base. Thus, during insufflation of root canal treatment, air will have entered the cervical fascia and cervical lodges. In the majority of the papers, recruited removal of the second and third molar teeth seems to be the main reason for mediastinal emphysema development in the dental practice.

**Fig. 4:**
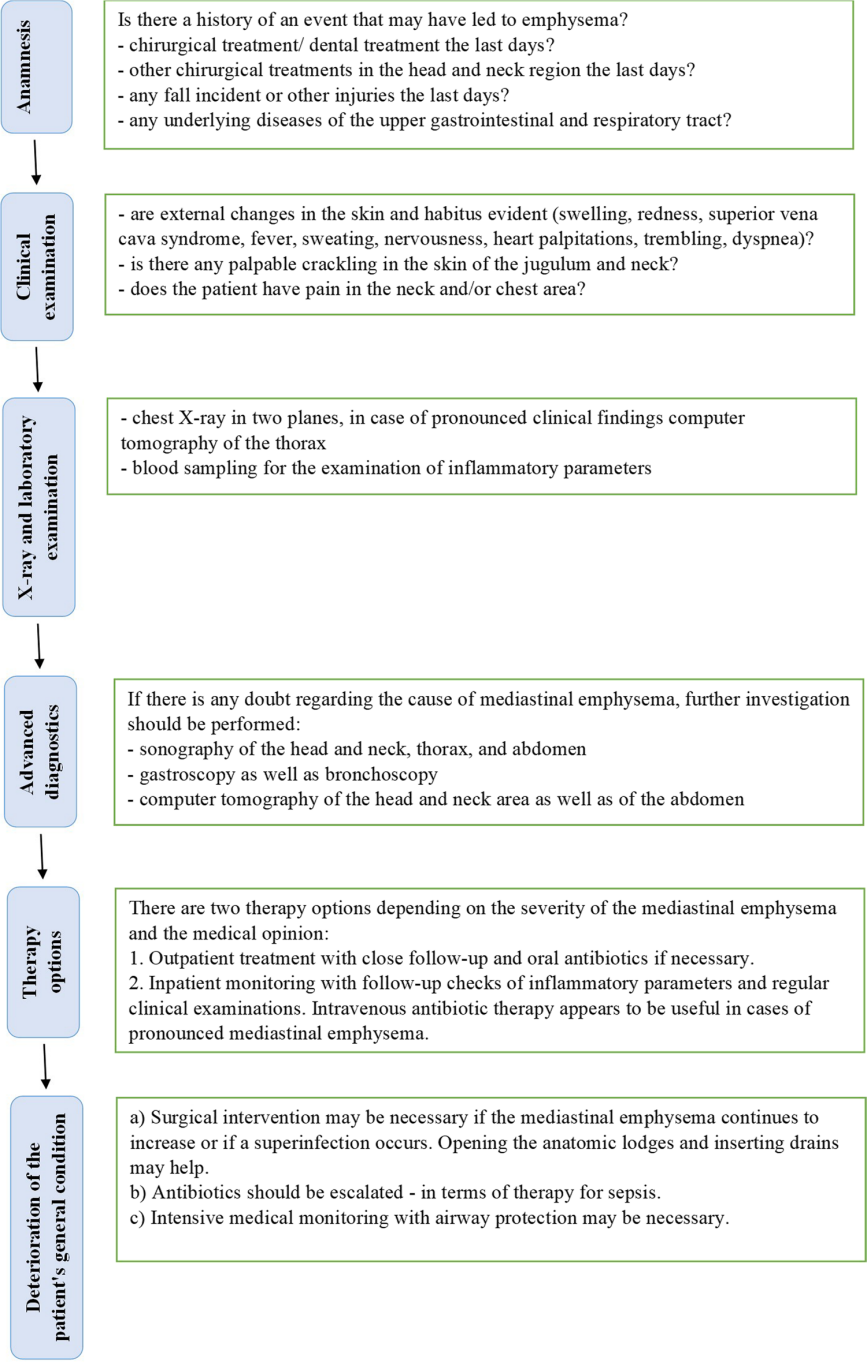
This diagram enables a guideline in medical diagnostics and therapy of the mediastinal emphysema, especially after dental treatments. Initially, a detailed anamnesis should be obtained to identify the probable cause of the mediastinal emphysema. The following clinical examination as well as the X-ray and laboratory diagnostic give an indication of the extent of the disease. If the cause of the mediastinal emphysema is still unclear, further medical examinations should be performed. In case of pronounced symptoms and clinical manifestation of the mediastinal emphysema, inpatient monitoring should be recommended

The subcutaneous emphysema may occur within minutes to hours after dental surgery. Erroneously, soft tissue emphysema is often misdiagnosed as an allergic reaction or acute postoperative swelling [24]. Subcutaneous emphysema and mediastinal emphysema usually do not have dental causes. More often are causes as traumatic intubation, mechanical ventilation, facial trauma, violent vomiting with rupture of the oesophagus, asthma exacerbation with alveolar rupture, or surgical procedures in the head and neck region [25]. Although our patient presented to a general practitioner because of the swelling, her medical history was incorrectly treated as a mild allergic reaction and cortisone was administered. A more detailed clinical examination and diagnosis could have ruled out an allergic reaction, especially since no other allergic symptoms such as cardio-respiratory problems occurred in the patient [26]. Angioedema, allergic reactions caused by the local anaesthetic, haematomas, and soft tissue infections are important differential diagnosis. Detailed history, clinical examination including palpable crepitations, and radiologic imaging can usually diagnose emphysema [27]. Figure 4 provides a guideline in the diagnosis of mediastinal emphysema.

The major limitation of this case report is that the computer tomography was performed on an emergency basis, whereby the representation of the teeth is only imprecise. The exact way of the roots of the second molar can only be estimated. For further diagnosis, a dental computed tomography would be necessary.

## Conclusion

This case report of pronounced facial and mediastinal emphysema after tooth preservation of the second molar in the lower jaw demonstrates how quickly an iatrogenic emphysema can be overlooked by treating professional physicians without further diagnosis. Soft tissue emphysema is a rare complication of dental treatment as root canal treatment or filling therapy, but it should always be considered as a differential diagnosis. Particularly soft tissue emphysema, especially those extending into the mediastinum, can present life-threatening complications. Moreover, the review of the literature shows developments of emphysema after different dental treatments, intending, to reduce the lack of knowledge of complications after dental treatments.

Thus, a medical consultation should always be made and the need for inpatient monitoring should be discussed. At least, the prescription of a prophylactic broad-spectrum antibiotic should be considered.

## Data Availability

The datasets generated during and analysed during the current study are available from the corresponding author on reasonable request.
